# Tetra­kis(2-amino-4-methyl­pyridinium) *cyclo*-tetra-μ_2_-oxido-tetra­kis­[dioxido­vanadate(V)] tetra­hydrate

**DOI:** 10.1107/S1600536811026912

**Published:** 2011-07-13

**Authors:** Masoumeh Tabatabaee, Ghasem Ahadiat, Krešimir Molčanov

**Affiliations:** a Department of Chemistry, Islamic Azad University, Yazd branch, Yazd, Iran; b Department of Physical Chemistry, Rudjer Bošković Institute, Bijenička 54, HR-10000 Zagreb, Croatia

## Abstract

The asymmetric unit of the title compound, (C_6_H_9_N_2_)_4_[V_4_O_12_]·4H_2_O, contains half of a [V_4_O_12_]^4−^ anion, two 2-amino-4-methyl­pyridinium, (2a4mpH)^+^, cations and two water mol­ecules. One water mol­ecule is disordered over two sets of sites with equal occupancies and the H atoms for this mol­ecule were not included in the refinement. The cation lies on an inversion center with four tetra­hedral VO_4_ units each sharing two vertices, forming an eight-membered ring. In the crystal, the components are linked by inter­molecular N—H⋯O hydrogen bonds, forming a one-dimensional network along [100]. Further stabilization is provided by weak inter­molecular C—H⋯O hydrogen bonds. In addition, π–π stacking inter­actions with centroid–centroid distances of 3.5420 (18), 3.7577 (18) and 3.6311 (19) Å are observed.

## Related literature

For related structures, see: Paredes-García *et al.* (2008) ▶; Nakano *et al.* (2002 ▶).
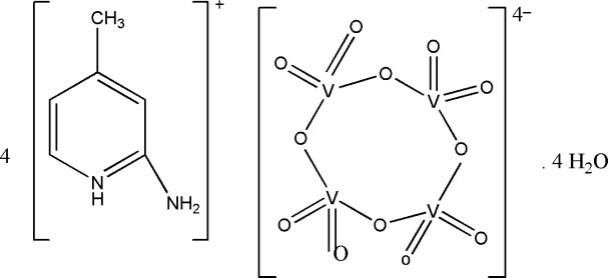


## Experimental

### 

#### Crystal data

(C_6_H_9_N_2_)_4_[V_4_O_12_]·4H_2_O
*M* *_r_* = 900.39
Triclinic, 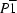

*a* = 7.8739 (3) Å
*b* = 11.1880 (5) Å
*c* = 11.7618 (6) Å
α = 73.609 (4)°
β = 76.945 (4)°
γ = 79.342 (4)°
*V* = 960.15 (7) Å^3^
*Z* = 1
Cu *K*α radiation
μ = 8.59 mm^−1^
*T* = 293 K
0.15 × 0.15 × 0.10 mm

#### Data collection

Oxford Diffraction Xcalibur Nova R diffractometer
Absorption correction: multi-scan (*CrysAlis PRO*; Oxford Diffraction, 2007 ▶) *T*_min_ = 0.518, *T*_max_ = 1
8031 measured reflections
3932 independent reflections
3371 reflections with *I* > 2σ(*I*)
*R*_int_ = 0.033

#### Refinement

*R*[*F* ^2^ > 2σ(*F* ^2^)] = 0.044
*wR*(*F* ^2^) = 0.129
*S* = 1.05
3932 reflections
250 parameters
3 restraints
H atoms treated by a mixture of independent and constrained refinement
Δρ_max_ = 0.47 e Å^−3^
Δρ_min_ = −0.33 e Å^−3^

### 

Data collection: *CrysAlis PRO* (Oxford Diffraction, 2007) ▶; cell refinement: *CrysAlis PRO*; data reduction: *CrysAlis PRO*; program(s) used to solve structure: *SHELXS86* (Sheldrick, 2008 ▶); program(s) used to refine structure: *SHELXL97* (Sheldrick, 2008 ▶); molecular graphics: *ORTEP-3 for Windows* (Farrugia, 1997 ▶) and *Mercury* (Macrae *et al.*, 2008 ▶); software used to prepare material for publication: *WinGX* (Farrugia, 1999) ▶.

## Supplementary Material

Crystal structure: contains datablock(s) global, I. DOI: 10.1107/S1600536811026912/lh5277sup1.cif

Structure factors: contains datablock(s) I. DOI: 10.1107/S1600536811026912/lh5277Isup2.hkl

Additional supplementary materials:  crystallographic information; 3D view; checkCIF report

## Figures and Tables

**Table 1:** Hydrogen-bond geometry (Å, °)

*D*—H⋯*A*	*D*—H	H⋯*A*	*D*⋯*A*	*D*—H⋯*A*
N1—H1⋯O1^i^	0.86	1.85	2.700 (3)	167
N2—H2*A*⋯O3^i^	0.86	2.00	2.861 (3)	178
N2—H2*B*⋯O2^ii^	0.86	2.13	2.959 (3)	161
N3—H3⋯O4^ii^	0.86	1.92	2.767 (3)	167
N4—H4*A*⋯O6^ii^	0.86	2.26	2.995 (4)	143
N4—H4*B*⋯O5^i^	0.86	2.04	2.883 (4)	165
C2—H2⋯O1^ii^	0.93	2.60	3.363 (4)	140
C2—H2⋯O2^ii^	0.93	2.64	3.371 (4)	136
C4—H4⋯O5	0.93	2.52	3.352 (4)	149

Symmetry codes: (i) 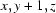; (ii) 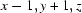.

**Fig. 1.**
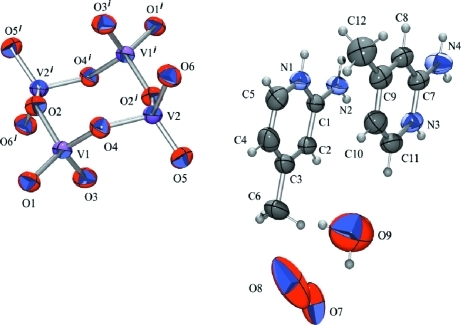
ORTEP-3 drawing of the asymmetric unit of the title compound with displacement ellipsoids drawn at the 50 % proabality leveland hydrogen atoms are depicted as spheres of arbitrary radii. Symmetry operator: (i) 2 - x, -y, 1 - z.

**Fig. 2.**
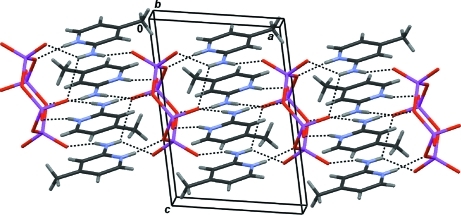
A hydrogen-bonded (dotted lines) chain consisting of anions and cations, extending in the direction [100]. The solvent water molecules are not shown.

**Fig. 3.**
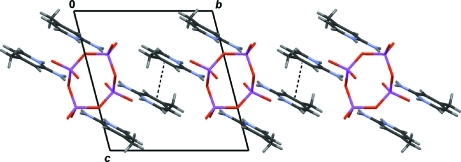
Packing of the title compound viewed along [100] with π–π stacking interactions shown as dashed lines.

## supplementary crystallographic information

### Comment

The chemistry of polyoxovanadate compounds are of great interest. Hybrid organo-inorganic compounds based on vanadium oxides present potential applications in catalysis, electron conductivity, magnetism and photochemistry (Paredes-García *et al.*, 2008). The VO_4_ group is an important building block of many polynuclear species. A well known example is the V_4_O_12_^4–^ ring which has an eight-membered ring structure formed by four VO_4_ tetrahedra sharing vertices (Nakano *et al.*, 2002). The complexing ability of the V_4_O_12_^4–^ ion with transition metal ions is of great interest and the ring takes part as a ligand (Paredes-García *et al.*, 2008). Herein we report the crystal structure of the title compound obtained as a side product from a reaction of ammonium vanadate, cobalt nitrate, boric acid and 2-Amino-4-methylpyridine.

The asymmetric unit of the title compound, (2a4mpH)_4_(V_4_O_12_).4H_2_O, comprises a half of a V_4_O_12_^4–^ anion, two (2a4mpH)^+^ cations and two solvent molecules of water (Fig. 1). One molecule of water is disordered over two positions (O7 and O8) with equal occupancies.

The V_4_O_12_^4–^ ion is centrosymmetric with four tetrahedral VO_4_units which share two vertices with each other to form an eight-membered ring. The 2-Amino-4-methylpyridine molecule is protonated *via* its endocyclic nitrogen atom. In the crystal, extensive intermolecular N—H···O hydrogen-bonding interactions (Table 1) between cations, anions and solvent water molecules form 1-D motive chains along [100] (Fig.2). The crystal packing is defined by a layered structure in which chains involving 2a4mpH^+^ and V_4_O_12_^4–^ ions are associated *via*π–π stacking interactions between the aromatic rings of (2a4mpH)^+^ cations into layers parallel to (110) (Fig. 3).

### Experimental

Ammonium vanadate, cobalt nitrate hexahydrate, boric acidand 2-amino-4-methylpyridine (in molar ratio 0.5:1:1:2) were dissolved in H_2_O (50ml). The reaction mixture was placed in a Parr-Teflon lined stainless steel vessel. It was sealed and heated at 443K for 48 h. The reaction mixture was gradually cooled to room temperature. Pale yellow crystals were isolated from solution.

### Refinement

A water molecule is disordered over two positions (O7 and O8) with equal occupancies. The H atoms for this molecule were not located nor were they included in the refinement. They are however, included in the molecular formula. Hydrogen atoms bound to the water molecule O9 were refined using the following restraints: O—H bond length 0.95 (2) Å and H···H distance 1.50 (4) Å. All other H atoms were placed in calculated positions with C-H = 0.93 - 0.96Å and N-H = 0.86Å and were included in the refinement with U_iso_(H) = 1.2U_eq_(C, N) or 1.5U_eq_(C_methyl_)

### Crystal data

**Table:**

(C_6_H_9_N_2_)_4_[V_4_O_12_]·4H_2_O	*Z* = 1
*M**_r_* = 900.39	*F*(000) = 332
Triclinic, *P*1	*D*_x_ = 1.557 Mg m^−^^3^
Hall symbol: -P 1	Cu *K*α radiation, λ = 1.54179 Å
*a* = 7.8739 (3) Å	Cell parameters from 4890 reflections
*b* = 11.1880 (5) Å	θ = 4.0–75.8°
*c* = 11.7618 (6) Å	µ = 8.59 mm^−^^1^
α = 73.609 (4)°	*T* = 293 K
β = 76.945 (4)°	Prism, pale yellow
γ = 79.342 (4)°	0.15 × 0.15 × 0.10 mm
*V* = 960.15 (7) Å^3^	

### Data collection

**Table:**

Oxford Diffraction Xcalibur Nova R diffractometer	3932 independent reflections
graphite	3371 reflections with *I* > 2σ(*I*)
Detector resolution: 10.4323 pixels mm^-1^	*R*_int_ = 0.033
ω scans	θ_max_ = 76.0°, θ_min_ = 4.0°
Absorption correction: multi-scan (*CrysAlis PRO*; Oxford Diffraction, 2007)	*h* = −9→9
*T*_min_ = 0.518, *T*_max_ = 1	*k* = −13→13
8031 measured reflections	*l* = −14→11

### Refinement

**Table:**

Refinement on *F*^2^	3 restraints
Least-squares matrix: full	H atoms treated by a mixture of independent and constrained refinement
*R*[*F*^2^ > 2σ(*F*^2^)] = 0.044	*w* = 1/[σ^2^(*F*_o_^2^) + (0.085*P*)^2^ + 0.0501*P*] where *P* = (*F*_o_^2^ + 2*F*_c_^2^)/3
*wR*(*F*^2^) = 0.129	(Δ/σ)_max_ = 0.001
*S* = 1.05	Δρ_max_ = 0.47 e Å^−^^3^
3932 reflections	Δρ_min_ = −0.33 e Å^−^^3^
250 parameters	

### Special details

**Table:**

Geometry. All e.s.d.'s (except the e.s.d. in the dihedral angle between two l.s. planes) are estimated using the full covariance matrix. The cell e.s.d.'s are taken into account individually in the estimation of e.s.d.'s in distances, angles and torsion angles; correlations between e.s.d.'s in cell parameters are only used when they are defined by crystal symmetry. An approximate (isotropic) treatment of cell e.s.d.'s is used for estimating e.s.d.'s involving l.s. planes.

### Fractional atomic coordinates and isotropic or equivalent isotropic displacement parameters (Å^2^)

**Table:**

	*x*	*y*	*z*	*U*_iso_*/*U*_eq_	Occ. (<1)
V2	0.96401 (5)	0.16480 (4)	0.30891 (4)	0.04115 (14)	
V1	0.95947 (5)	−0.13283 (4)	0.42124 (4)	0.03986 (14)	
O1	0.9714 (3)	−0.24965 (19)	0.3592 (2)	0.0530 (5)	
O2	1.0932 (2)	−0.17853 (19)	0.53675 (18)	0.0498 (4)	
O3	0.7553 (3)	−0.0950 (2)	0.4789 (2)	0.0589 (5)	
O4	1.0430 (2)	0.00128 (17)	0.30665 (17)	0.0473 (4)	
O5	0.7916 (3)	0.2148 (2)	0.2444 (2)	0.0575 (5)	
O6	1.1255 (3)	0.2451 (2)	0.2325 (2)	0.0600 (5)	
C1	0.5148 (3)	0.6860 (2)	0.4084 (2)	0.0446 (5)	
C2	0.3830 (4)	0.6228 (3)	0.3964 (3)	0.0489 (6)	
H2	0.2663	0.645	0.4292	0.059*	
C3	0.4245 (5)	0.5298 (3)	0.3373 (3)	0.0577 (7)	
C4	0.6019 (5)	0.4953 (3)	0.2905 (3)	0.0647 (8)	
H4	0.6333	0.4311	0.2509	0.078*	
C5	0.7261 (4)	0.5561 (3)	0.3034 (3)	0.0620 (7)	
H5	0.8432	0.534	0.2714	0.074*	
C6	0.2802 (6)	0.4652 (4)	0.3238 (4)	0.0836 (12)	
H6C	0.3308	0.4022	0.2805	0.125*	
H6A	0.1994	0.526	0.2801	0.125*	
H6B	0.2188	0.4264	0.4021	0.125*	
N1	0.6839 (3)	0.6492 (2)	0.3625 (2)	0.0497 (5)	
H1	0.7662	0.6853	0.3708	0.06*	
N2	0.4799 (3)	0.7794 (2)	0.4625 (2)	0.0547 (6)	
H2A	0.5642	0.8155	0.4679	0.066*	
H2B	0.3731	0.8038	0.4921	0.066*	
C7	0.5200 (4)	1.0124 (3)	0.2050 (3)	0.0523 (6)	
C8	0.6928 (4)	0.9673 (3)	0.1585 (3)	0.0521 (6)	
H8	0.7863	1.0016	0.1677	0.062*	
C9	0.7251 (4)	0.8738 (3)	0.1000 (3)	0.0563 (7)	
C10	0.5819 (5)	0.8220 (3)	0.0874 (3)	0.0622 (7)	
H10	0.6011	0.7592	0.0466	0.075*	
C11	0.4163 (4)	0.8645 (3)	0.1352 (3)	0.0601 (7)	
H11	0.3217	0.8295	0.1287	0.072*	
C12	0.9095 (5)	0.8251 (4)	0.0517 (4)	0.0783 (10)	
H12B	0.9078	0.7602	0.0133	0.117*	
H12A	0.9734	0.7914	0.1167	0.117*	
H12C	0.9656	0.8924	−0.0061	0.117*	
N3	0.3879 (3)	0.9573 (3)	0.1922 (2)	0.0559 (6)	
H3	0.2815	0.9827	0.2218	0.067*	
N4	0.4820 (4)	1.1037 (3)	0.2601 (3)	0.0750 (8)	
H4A	0.3743	1.1281	0.2878	0.09*	
H4B	0.5649	1.1391	0.2686	0.09*	
O9	0.3335 (12)	0.6497 (8)	−0.0146 (6)	0.186 (3)	
O7	0.0661 (13)	0.4852 (7)	0.0806 (8)	0.121 (3)	0.5
O8	0.754 (2)	0.5676 (10)	−0.0400 (7)	0.191 (7)	0.5
H9A	0.255 (15)	0.616 (10)	−0.047 (11)	0.3*	
H9B	0.385 (17)	0.573 (7)	0.037 (10)	0.3*	

### Atomic displacement parameters (Å^2^)

**Table:**

	*U*^11^	*U*^22^	*U*^33^	*U*^12^	*U*^13^	*U*^23^
V2	0.0395 (2)	0.0430 (2)	0.0433 (2)	−0.00904 (16)	−0.01011 (16)	−0.01024 (17)
V1	0.0361 (2)	0.0433 (2)	0.0449 (2)	−0.00975 (15)	−0.00796 (16)	−0.01537 (17)
O1	0.0542 (10)	0.0535 (10)	0.0622 (12)	−0.0098 (8)	−0.0159 (9)	−0.0259 (9)
O2	0.0471 (9)	0.0566 (10)	0.0500 (10)	−0.0079 (8)	−0.0129 (8)	−0.0164 (8)
O3	0.0415 (9)	0.0704 (13)	0.0701 (13)	−0.0124 (8)	−0.0044 (9)	−0.0273 (11)
O4	0.0453 (9)	0.0458 (9)	0.0518 (10)	−0.0089 (7)	−0.0060 (7)	−0.0141 (8)
O5	0.0538 (11)	0.0611 (12)	0.0635 (12)	−0.0017 (9)	−0.0250 (9)	−0.0175 (10)
O6	0.0578 (11)	0.0601 (12)	0.0615 (12)	−0.0238 (9)	−0.0071 (9)	−0.0069 (10)
C1	0.0447 (12)	0.0455 (12)	0.0445 (12)	−0.0119 (9)	−0.0129 (10)	−0.0050 (10)
C2	0.0446 (12)	0.0478 (13)	0.0556 (15)	−0.0120 (10)	−0.0164 (11)	−0.0057 (11)
C3	0.0701 (17)	0.0508 (14)	0.0603 (17)	−0.0165 (13)	−0.0298 (14)	−0.0074 (13)
C4	0.082 (2)	0.0559 (16)	0.0640 (18)	−0.0073 (15)	−0.0203 (16)	−0.0231 (14)
C5	0.0563 (16)	0.0669 (18)	0.0613 (18)	−0.0024 (13)	−0.0081 (13)	−0.0192 (15)
C6	0.103 (3)	0.071 (2)	0.098 (3)	−0.033 (2)	−0.049 (2)	−0.018 (2)
N1	0.0434 (11)	0.0548 (12)	0.0530 (13)	−0.0138 (9)	−0.0096 (9)	−0.0113 (10)
N2	0.0443 (11)	0.0592 (13)	0.0667 (15)	−0.0142 (9)	−0.0080 (10)	−0.0224 (12)
C7	0.0422 (13)	0.0661 (16)	0.0475 (14)	−0.0106 (11)	−0.0094 (10)	−0.0095 (12)
C8	0.0453 (13)	0.0668 (17)	0.0436 (13)	−0.0120 (11)	−0.0086 (10)	−0.0097 (12)
C9	0.0547 (15)	0.0694 (18)	0.0410 (13)	−0.0093 (13)	−0.0070 (11)	−0.0086 (12)
C10	0.0710 (19)	0.0665 (18)	0.0530 (16)	−0.0120 (15)	−0.0173 (14)	−0.0147 (14)
C11	0.0597 (16)	0.0683 (18)	0.0555 (16)	−0.0216 (14)	−0.0220 (13)	−0.0033 (14)
C12	0.066 (2)	0.097 (3)	0.069 (2)	−0.0061 (18)	0.0031 (17)	−0.032 (2)
N3	0.0404 (11)	0.0698 (15)	0.0544 (13)	−0.0123 (10)	−0.0082 (9)	−0.0077 (11)
N4	0.0472 (13)	0.090 (2)	0.097 (2)	−0.0088 (13)	−0.0057 (14)	−0.0436 (19)
O9	0.225 (8)	0.230 (8)	0.116 (4)	−0.091 (6)	−0.042 (4)	−0.016 (5)
O7	0.161 (8)	0.072 (4)	0.101 (6)	−0.012 (4)	−0.004 (5)	0.007 (4)
O8	0.38 (2)	0.118 (7)	0.071 (5)	−0.122 (11)	0.017 (8)	−0.006 (5)

### Geometric parameters (Å, °)

**Table:**

V2—O5	1.636 (2)	N2—H2A	0.86
V2—O6	1.637 (2)	N2—H2B	0.86
V2—O2^i^	1.812 (2)	C7—N4	1.314 (4)
V2—O4	1.8258 (18)	C7—N3	1.358 (4)
V1—O3	1.625 (2)	C7—C8	1.404 (4)
V1—O1	1.6467 (19)	C8—C9	1.364 (5)
V1—O2	1.809 (2)	C8—H8	0.93
V1—O4	1.8232 (19)	C9—C10	1.414 (5)
O2—V2^i^	1.812 (2)	C9—C12	1.495 (5)
C1—N2	1.328 (4)	C10—C11	1.353 (5)
C1—N1	1.353 (4)	C10—H10	0.93
C1—C2	1.411 (4)	C11—N3	1.349 (5)
C2—C3	1.361 (4)	C11—H11	0.93
C2—H2	0.93	C12—H12B	0.96
C3—C4	1.406 (5)	C12—H12A	0.96
C3—C6	1.513 (4)	C12—H12C	0.96
C4—C5	1.346 (5)	N3—H3	0.86
C4—H4	0.93	N4—H4A	0.86
C5—N1	1.362 (4)	N4—H4B	0.86
C5—H5	0.93	O9—H9A	0.97 (2)
C6—H6C	0.96	O9—H9B	0.98 (2)
C6—H6A	0.96	O7—O8^ii^	1.460 (18)
C6—H6B	0.96	O8—O7^ii^	1.460 (18)
N1—H1	0.86		
			
O5—V2—O6	109.93 (12)	C1—N1—C5	121.2 (3)
O5—V2—O2^i^	109.51 (10)	C1—N1—H1	119.4
O6—V2—O2^i^	111.16 (11)	C5—N1—H1	119.4
O5—V2—O4	110.27 (10)	C1—N2—H2A	120
O6—V2—O4	106.15 (10)	C1—N2—H2B	120
O2^i^—V2—O4	109.79 (9)	H2A—N2—H2B	120
O3—V1—O1	108.79 (11)	N4—C7—N3	119.4 (3)
O3—V1—O2	110.32 (11)	N4—C7—C8	123.0 (3)
O1—V1—O2	110.20 (10)	N3—C7—C8	117.5 (3)
O3—V1—O4	110.02 (11)	C9—C8—C7	120.8 (3)
O1—V1—O4	109.41 (10)	C9—C8—H8	119.6
O2—V1—O4	108.10 (9)	C7—C8—H8	119.6
V1—O2—V2^i^	129.30 (11)	C8—C9—C10	119.0 (3)
V1—O4—V2	123.94 (10)	C8—C9—C12	120.7 (3)
N2—C1—N1	118.9 (2)	C10—C9—C12	120.3 (3)
N2—C1—C2	122.9 (3)	C11—C10—C9	119.4 (3)
N1—C1—C2	118.2 (3)	C11—C10—H10	120.3
C3—C2—C1	120.8 (3)	C9—C10—H10	120.3
C3—C2—H2	119.6	N3—C11—C10	120.4 (3)
C1—C2—H2	119.6	N3—C11—H11	119.8
C2—C3—C4	118.9 (3)	C10—C11—H11	119.8
C2—C3—C6	119.7 (3)	C9—C12—H12B	109.5
C4—C3—C6	121.4 (3)	C9—C12—H12A	109.5
C5—C4—C3	119.5 (3)	H12B—C12—H12A	109.5
C5—C4—H4	120.3	C9—C12—H12C	109.5
C3—C4—H4	120.3	H12B—C12—H12C	109.5
C4—C5—N1	121.4 (3)	H12A—C12—H12C	109.5
C4—C5—H5	119.3	C11—N3—C7	122.8 (3)
N1—C5—H5	119.3	C11—N3—H3	118.6
C3—C6—H6C	109.5	C7—N3—H3	118.6
C3—C6—H6A	109.5	C7—N4—H4A	120
H6C—C6—H6A	109.5	C7—N4—H4B	120
C3—C6—H6B	109.5	H4A—N4—H4B	120
H6C—C6—H6B	109.5	H9A—O9—H9B	102 (4)
H6A—C6—H6B	109.5		
			
O3—V1—O2—V2^i^	−9.82 (19)	C3—C4—C5—N1	0.9 (5)
O1—V1—O2—V2^i^	−129.97 (15)	N2—C1—N1—C5	−178.3 (3)
O4—V1—O2—V2^i^	110.52 (14)	C2—C1—N1—C5	1.7 (4)
O3—V1—O4—V2	29.34 (16)	C4—C5—N1—C1	−1.3 (5)
O1—V1—O4—V2	148.80 (13)	N4—C7—C8—C9	−178.8 (3)
O2—V1—O4—V2	−91.18 (13)	N3—C7—C8—C9	1.7 (5)
O5—V2—O4—V1	−86.26 (15)	C7—C8—C9—C10	−0.5 (5)
O6—V2—O4—V1	154.71 (13)	C7—C8—C9—C12	−179.4 (3)
O2^i^—V2—O4—V1	34.48 (15)	C8—C9—C10—C11	−1.1 (5)
N2—C1—C2—C3	178.2 (3)	C12—C9—C10—C11	177.8 (3)
N1—C1—C2—C3	−1.8 (4)	C9—C10—C11—N3	1.4 (5)
C1—C2—C3—C4	1.4 (5)	C10—C11—N3—C7	0.0 (5)
C1—C2—C3—C6	−178.9 (3)	N4—C7—N3—C11	179.0 (3)
C2—C3—C4—C5	−1.0 (5)	C8—C7—N3—C11	−1.5 (4)
C6—C3—C4—C5	179.3 (4)		

Symmetry codes: (i) −*x*+2, −*y*, −*z*+1; (ii) −*x*+1, −*y*+1, −*z*.

### Hydrogen-bond geometry (Å, °)

**Table:**

*D*—H···*A*	*D*—H	H···*A*	*D*···*A*	*D*—H···*A*
N1—H1···O1^iii^	0.86	1.85	2.700 (3)	167
N2—H2A···O3^iii^	0.86	2.00	2.861 (3)	178
N2—H2B···O2^iv^	0.86	2.13	2.959 (3)	161
N3—H3···O4^iv^	0.86	1.92	2.767 (3)	167
N4—H4A···O6^iv^	0.86	2.26	2.995 (4)	143
N4—H4B···O5^iii^	0.86	2.04	2.883 (4)	165
C2—H2···O1^iv^	0.93	2.60	3.363 (4)	140
C2—H2···O2^iv^	0.93	2.64	3.371 (4)	136
C4—H4···O5	0.93	2.52	3.352 (4)	149

Symmetry codes: (iii) *x*, *y*+1, *z*; (iv) *x*−1, *y*+1, *z*.
